# Adipose Tissue-Derived Stromal Cells Induce a Highly Trophic Environment While Reducing Maturation of Monocyte-Derived Dendritic Cells

**DOI:** 10.1155/2020/8868909

**Published:** 2020-10-26

**Authors:** Morten Juhl, Bjarke Follin, Monika Gad, Jesper Larsen, Jens Kastrup, Annette Ekblond

**Affiliations:** ^1^Cardiology Stem Cell Centre, The Centre for Cardiac, Vascular, Pulmonary and Infectious Diseases, Copenhagen University Hospital Rigshospitalet, 2100 Copenhagen, Denmark; ^2^Bioneer A/S, 2970 Hørsholm, Denmark

## Abstract

Allogeneic cell-based therapies using adipose tissue-derived stromal cells (ASCs) offer an off-the-shelf alternative to autologous therapy. An underlying assumption is that ASC can modulate the immune response of the recipient. However, *in vitro* models are required to explore and identify cell interactions and mechanisms of action, to ensure sufficient and sustained effects, and to document these. In this study, we shed light on the effect of ASC manufactured for clinical use on monocyte-derived dendritic cells and an inflammatory microenvironment. ASCs were isolated from healthy voluntary donors, expanded using a human platelet lysate in bioreactors, and cryopreserved as per clinical use. Monocyte-derived dendritic cells were generated by isolation of monocytes and differentiation with GM-CSF and IL-4. Dendritic cells were cocultured with different ratios of ASC and matured with LPS and IFN-*γ*. Dexamethasone was included as an immunosuppressive control. Dendritic cells were analyzed by flow cytometry for CD11c, CD40, CD80, CD83, CD86, PD-L1, and HLA-DR, and supernatants were analyzed for FGF2, HGF, IL-10, IL-12p70, LIF, MIF, PDGF, PlGF, and IDO. Reduced expression of maturation markers was observed on ASC-treated dendritic cells, while high levels of PD-L1 were maintained. Interestingly, the expression of CD83 was elevated. Escalating ratios of ASC did not affect the concentration of IL-10 considerably, whereas the presence of IL-12 was reduced in a dose-dependent manner. Besides offsetting the IL-12/IL-10 balance, the concentrations of IDO and MIF were elevated in cocultures. Concentrations of FGF2, HGF, LIF, and PIGF were high in ASC cocultures, whereas PDGF was depleted. In a robust coculture model, the addition of ASC to dendritic cells inhibited the dendritic maturation substantially, while inducing a less inflammatory and more tolerogenic milieu. Despite the exposure to dendritic cells and inflammatory stimuli, ASC resulted in supernatants with trophic factors relevant for regeneration. Thus, ASC can perform immunomodulation while providing a regenerative environment.

## 1. Introduction

Mesenchymal stromal cells (MSC) have emerged as a promising source for therapy, as these cells not only possess a sound regenerative potential but also have favorable immunological properties, rendering allogeneic therapy a viable option [1]. At the Cardiology Stem Cell Centre, we have developed Good Manufacturing Procedure methods for production of MSC derived from adipose tissue, the so-called adipose tissue-derived stromal cells (ASCs), for allogeneic therapy [2]. Besides demonstrating low immunogenicity, evidence suggests that if conditions are optimal, MSC and ASC are capable of immunomodulation, which expands the therapeutic potential considerably [3]. The immunomodulation has been shown to function on several immune cell types, including the dendritic cell. Dendritic cells (DCs) constitute a critical component in mounting an adaptive response to foreign antigens, by bridging the innate and adaptive immune system, not directly eradicating pathogens, but conveying information and initiating persistent antigen-specific responses [4]. A growing body of evidence states that DCs act as regulators of immunity that, besides the ability to commence an adaptive response, are able to regulate central and peripheral immunity and induce tolerance [5]. Therefore, it is central to thoroughly characterize and understand this important interaction. In addition to the implications on the immune response, the microenvironment created by the interaction must also be able to support regeneration, since the main application of ASC treatment is for regenerative medicine. This has not previously been investigated in an ASC and DC coculture.

In the present study, we sought to thoroughly investigate how ASCs affect the maturation of DC and characterize the resulting DC phenotype and milieu. In doing so, we assayed several factors: some are new to our ASC cell product and are not previously sampled in a DC model. This information will aid the identification of ASC specific modes of action, and the rigorous model characterization will ease the future development of robust assays for functional characterization and quality control of clinical ASC products, also known as potency assays. Potency assays are desired to ensure proper efficacy of cell products, yet to fully resemble biological effects related to the therapeutic outcome, relevant functions and mechanisms of actions must be elucidated. Besides contributing to potency assays, documenting the effect of ASC on a critical immune cell type makes a compelling argument for allogeneic ASC therapy.

## 2. Materials and Methods

### 2.1. Adipose Tissue-Derived Stromal Cells

ASCs were produced as previously described [2, 6]. In brief, lipoaspirates were collected from eligible healthy donors upon written informed consent. Research on biological samples has been approved by the regional scientific ethical committee, Capital Region of Denmark. The stromal vascular fraction was isolated by enzymatic digestion and expanded for two passages using a semiautomated Quantum Cell Expansion System (Terumo BCT) in growth medium consisting of MEM-*α* (Gibco), 100 U/ml penicillin and 100 *μ*g/ml streptomycin (Gibco), and 5% Stemulate human platelet lysate (Cook Regentec). For later in vitro experimentation, several samples were seeded in cell culture-treated T175 flasks (Nunc) and expanded for an additional passage in growth medium. The resulting ASC population was harvested with TrypLE Select (Gibco) and centrifuged at 300g for 5 min at room temperature (RT). The pellet was resuspended in CryoStor CS10 (BioLife Solutions) at a concentration of 10^6^ cells/ml, aliquoted at 1 ml per vial in cryotubes (Nunc), and cryopreserved at a controlled -1°C/min rate using CoolCell FTS30 (BioCision) before transfer to storage in liquid nitrogen. Four days prior to use for experimental setups, vials of ASC were thawed in a 37°C water bath, diluted in growth medium, and seeded in T175 flasks to recover. A total of 5 ASC donors were included in the present study. The ASC product has previously been characterized in detail [6, 7].

### 2.2. Isolation of CD14+ Monocytes

Buffy coats were produced from healthy blood donors by the Department of Clinical Immunology, University Hospital Rigshospitalet, Copenhagen, Denmark, according to current rules for blood donation. Buffy coats were stored overnight at RT and diluted to 1 : 4 in PBSE buffer (DPBS without calcium and magnesium (Lonza), 2 mM EDTA (Merck), and 0.5% human AB serum (Valley Biomedical)) and layered on Ficoll-Paque (GE Healthcare) in Leucosep tubes (Greiner Bio-One). The tubes were centrifuged at 1000g for 10 minutes at RT, and the peripheral blood mononuclear cells were harvested and washed in PBSE followed by centrifugation. Unless otherwise stated, any subsequent centrifugation was performed at 300g for 5 min at RT. Washing was repeated twice. Cells were resuspended in degassed PBSE and filtered through a 70 *μ*m cell strainer (Falcon) before counting on a NucleoCounter NC-200 (Chemometec), and cell concentration was adjusted to 10^8^ cells/ml. For magnetic-activated cell sorting (MACS), CD14 MicroBeads (Miltenyi Biotec) were used according to the manufacturer's instructions.

### 2.3. Generation of Monocyte-Derived Dendritic Cells

The isolated monocytes were washed in complete medium (RPMI 1640 (Lonza), 100 U/ml penicillin and 100 *μ*g/ml streptomycin (Lonza), 2 mM L-glutamine (Lonza), and 5% human AB serum) and centrifuged prior to setup. Cells were counted and adjusted to a concentration of 2 × 10^6^ cells/ml in complete medium containing 20 ng/ml GM-CSF and 20 ng/ml IL-4 (PeproTech) before seeding 3 ml/well in six-well plates (Nunc) and incubation at 37°C and 5% CO_2_ humid conditions. After 3 days, medium was replaced and cells in a suspension were centrifuged and returned to culture. After 6 days of culture, the resulting immature dendritic cells (iDC) were harvested and used for experimentation. A total of 4 DC donors were included.

### 2.4. Experimental Setup

**Figure 1:**
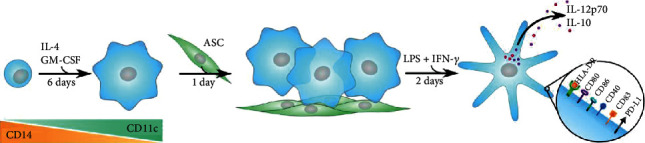
Experimental overview. Monocytes were isolated based on CD14 expression and induced to differentiate into immature dendritic cells by IL-4 and GM-CSF. Following 6 days of differentiation, small irregular dendrites were present. At this point, cocultures with ASC were established and allowed to recover for 1 day. The cocultures were matured by stimulation with LPS and IFN-*γ* for 2 days, supernatants were harvested and sampled for IL-12p70 and IL-10, and mature dendritic cells were analyzed for surface markers CD11c, HLA-DR, CD80, CD86, CD40, CD83, and PD-L1.

The iDC were adjusted to 10^5^ cells per well in round-bottom, surface-treated 96-well plates (Nunc) and seeded in quadruplicate, to which ASCs were added in ratios of 1 : 5, 1 : 10, and 1 : 20, besides controls devoid of ASC. As an additional control, 1 *μ*M dexamethasone (Sigma-Aldrich) was included. Cells were allowed to recover overnight, followed by the addition of a stimulating cocktail consisting of 0.1 *μ*g/ml LPS (Sigma-Aldrich) and 20 ng/ml IFN-*γ* (PeproTech) to generate mature dendritic cells (mDCs) as previously described [8]. After 48 hours, plates were centrifuged at 100g for 5 minutes at RT and supernatants were collected and stored at -80°C. 200 *μ*l cold PBSE was added per well to the cell pellets, and plates were incubated at 4°C to promote detachment for 10 minutes and gently resuspended with a pipette. All ASC donors were tested against each DC donor, resulting in a total of 20 combinations. An overview is illustrated in Figure 1.

### 2.5. Flow Cytometry

**Figure 2:**
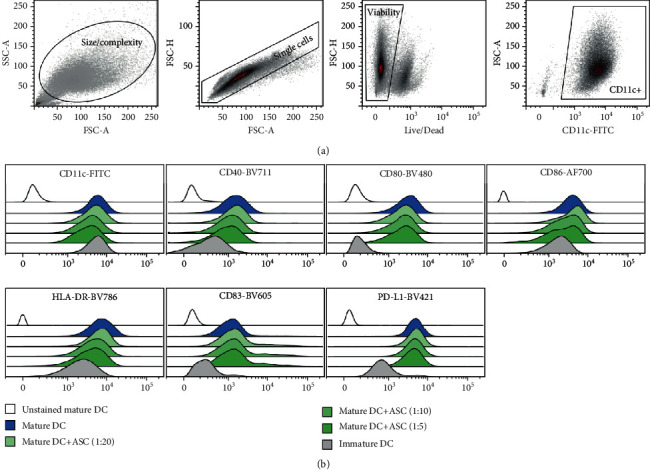
(a) Gating strategy. Sequential gates were set up based on size/complexity, single cells, viability, and CD11c+ events. Analysis of markers was performed on CD11c+ events. (b) Expression of markers. Histograms from a single representative DC donor and ASC donor in different ratios (ASC : DC ratio from 1 : 20 to 1 : 5).

Discrimination of viable cells was based on staining with the Live/Dead Fixable Near-IR Dead Cell Stain Kit (Thermo Fisher Scientific) according to the manufacturer's instructions. Following Live/Dead staining, pellets were resuspended in Brilliant Staining Buffer (BD Biosciences), treated with the FcR blocking reagent (Miltenyi Biotec), and stained with a multicolor antibody panel consisting of CD11c-FITC, CD40-BV711, CD80-BV480, CD83-BV605, CD86-AF700, PD-L1-BV421, and HLA-DR-BV786 (BD Biosciences). Samples were acquired on a FACSAria III flow cytometer (BD Biosciences) with automatic compensation based on single-stained mDC and analyzed in FlowLogic (Inivai Technologies). At least 25,000 CD11c+ events were recorded. The gating strategy was based on size/complexity (FSC/SSC), singlet discrimination (FSC-area/FSC-height), viability (APC-Cy7-negative), and CD11c+ (FITC) as illustrated in Figure 2. The median fluorescent intensity (MFI) was measured on CD11c+ cells, to exclude them possibly from ASCs that are CD11c-negative. The MFI was normalized to the intensity of the mDC phenotype of the given donor.

### 2.6. Cytokine Measurements

A custom premixed Luminex assay was acquired for FGF2, HGF, IL-10, IL-12p70, LIF, MIF, PDGF, and PlGF (R&D Systems), performed according to the manufacturer's instructions, and analyzed on a MAGPIX instrument (Luminex Corporation). IDO was assayed by ELISA (R&D Systems) according to the manufacturer's specifications, absorbance at 450 nm was read on a FLUOstar Omega microplate reader (BMG Labtech) with background subtraction at 540 nm, and concentrations were determined by 5-parameter logistic curve fit.

### 2.7. Statistical Analysis

Analysis was performed using nonparametric paired Wilcoxon signed-rank tests, as data contained outliers. Values from each group were compared to the values from the mDC monoculture with the same mDC donor run on the same plate during sample acquisition. Significance levels were set to *p* ≤ 0.05, and results were adjusted using the Bonferroni correction for multiple comparisons. Data is presented in box plots (Tukey) with significant differences from mDC highlighted with an asterisk (∗). Correlations were performed using Spearman's rho. All statistical analyses were performed using 6IBM SPSS Statistics (ver. 25, IBM Corporation). A correlation matrix was generated using R version 4.0.2 with the corrplot package [9].

## 3. Results

### 3.1. Adipose Tissue-Derived Stromal Cells Alter the Phenotypical Profile of Dendritic Cells

**Figure 3:**
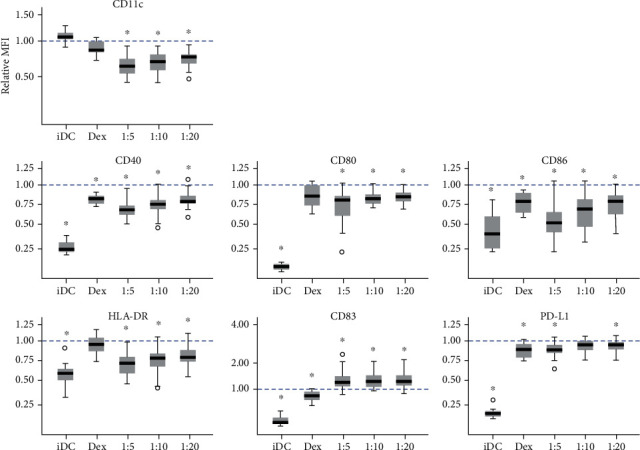
Expression of surface markers on dendritic cells. The dotted line indicates the level of mDC (1). 1 : 5-1 : 20 refer to ratios of ASC : DC. iDC: immature dendritic cell; Dex: dexamethasone control; mDC: mature dendritic cell; ASC: adipose tissue-derived stromal cell. Significant differences from mDC (adjusted *p* ≤ 0.05) marked with an asterisk (∗). *n* = 20, resulting from 5 ASC donors and 4 DC donors.

Upon activation and coculture with ASC, DC expression of CD11c was reduced (Figure 3). While the relative MFI of iDC compared to mDC (1.08 ± 0.03) and dexamethasone control (0.89 ± 0.04) remained high, the reduction of ASC cocultures was significant from 1 : 20 (0.72 ± 0.03) and 1 : 10 (0.68 ± 0.03) to 1 : 5 (0.63 ± 0.03). The relative MFI of hallmark DC markers CD40, CD80, CD86, and HLA-DR was lowered with increasing doses of ASC indicating a dose response. For CD40, the intensity dropped from 1 : 20 (0.79 ± 0.03) and 1 : 10 (0.74 ± 0.03) to 1 : 5 (0.68 ± 0.03), surpassing the effect of the dexamethasone control (0.80 ± 0.02), while iDC remained low (0.26 ± 0.02). The reduction in CD80 was less evident yet significantly lowered from mDC at all ratios of 1 : 20 (0.82 ± 0.02), 1 : 10 (0.80 ± 0.02), and 1 : 5 (0.71 ± 0.05). The CD80 expression on the dexamethasone control (0.85 ± 0.04) did not differ significantly from mDC, whereas iDC was very low (0.09 ± 0.01). The relative expression of CD86 fell from 1 : 20 (0.73 ± 0.04) to 1 : 10 (0.66 ± 0.04) and even to 1 : 5 (0.54 ± 0.04), with iDC (0.43 ± 0.06) being lower and the dexamethasone control (0.75 ± 0.04) being higher than ASC-treated cultures. Finally, HLA-DR expression on the dexamethasone control (0.95 ± 0.04) was not significantly different from that on mDC, while iDC displayed approximately half the level (0.57 ± 042). The addition of ASC reduced the expression of HLA-DR towards iDC, from 1 : 20 (0.79 ± 0.03) and 1 : 10 (0.73 ± 0.03) to 1 : 5 (0.68 ± 0.03).

In contrast to this, the expression of CD83 followed a reciprocal pattern. While the expression on iDC (0.24 ± 0.03) was low and the dexamethasone control (0.80 ± 0.04) decreased compared to mDC, the ratios of ASC caused elevated expression levels of 1 : 20 (1.27 ± 0.07), 1 : 10 (1.31 ± 0.07), and 1 : 5 (1.31 ± 0.08). PD-L1 intensity was significantly reduced on coculture, but the reduction was limited across conditions of the dexamethasone control (0.86 ± 0.03): 1 : 20 (0.93 ± 0.02), 1 : 10 (0.93 ± 0.02), and 1 : 5 (0.87 ± 0.02). The expression level of PD-L1 was low on iDC (0.17 ± 0.01).

### 3.2. A Less Inflammatory, More Suppressive Secretory Milieu upon ASC Cocultivation

**Figure 4:**
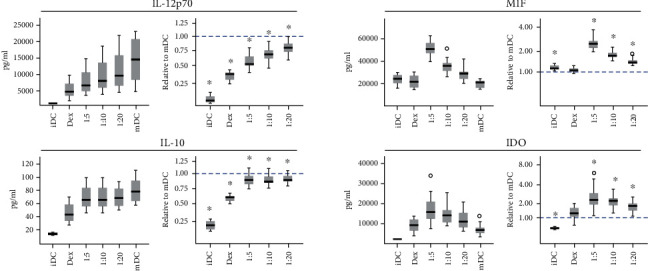
Absolute and relative concentrations of IL-12p70, IL-10, MIF, and IDO. 1 : 5-1 : 20 refer to ratios of ASC : DC. iDC: immature dendritic cell; Dex: dexamethasone control; mDC: mature dendritic cell; ASC: adipose tissue-derived stromal cell. Significant differences from mDC (adjusted *p* ≤ 0.05) marked with an asterisk (∗). *n* = 20, resulting from 5 ASC donors and 4 DC donors.

The addition of increasing doses of ASC to cocultures caused a significant reduction in interleukin 12 (IL-12p70) compared to mDC (Figure 4, Supplementary Materials table 1). Despite considerable DC donor variation, the concentrations relative to mDC were affected similarly by the addition of ASC at ratios of 1 : 20 (0.78 ± 0.02), 1 : 10 (0.68 ± 0.02), and 1 : 5 (0.55 ± 0.02), which were surpassed by the dexamethasone control (0.35 ± 0.02). iDC concentrations were very low (0.07 ± 0.01).

The concentration of interleukin 10 (IL-10) did not change substantially in response to ASC, but remained steady, whereas the dexamethasone control significantly lowered the concentration. The concentration for iDC (0.20 ± 0.02) was low, as was that for the dexamethasone control (0.58 ± 0.02). The addition of ASC resulted in comparable high levels at 1 : 20 (0.89 ± 0.02), 1 : 10 (0.88 ± 0.02), and 1 : 5 (0.89 ± 0.02), but such level was not higher than the level for mDC alone.

A significant increase in the macrophage migration inhibitory factor (MIF) and indoleamine 2,3-dioxygenase (IDO) was detected in ASC-containing cultures, and the effect of escalating ratios suggests a dose response. The concentration of MIF was higher in iDC (1.19 ± 0.04) and insignificant in comparison to mDC in the dexamethasone control (1.09 ± 0.03), yet heavily exceeded by 1 : 20 (1.45 ± 0.04), 1 : 10 (1.82 ± 0.05), and 1 : 5 (2.61 ± 0.09). A similar pattern presented itself for IDO, which was elevated but insignificant in the dexamethasone control (1.33 ± 0.12) and surpassed by 1 : 20 (1.81 ± 0.08), 1 : 10 (2.22 ± 0.11), and 1 : 5 (2.74 ± 0.27). iDC was considerably lower (0.51 ± 0.02).

### 3.3. Enhanced Trophic Environment in ASC Cocultures

**Figure 5:**
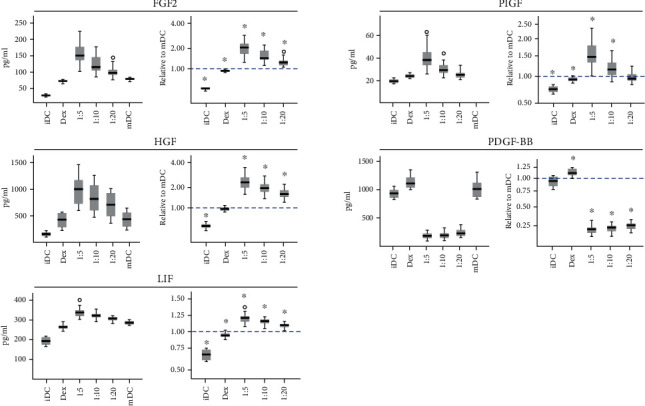
Absolute and relative concentrations of FGF2, HGF, LIF, PlGF, and PDGF-BB. 1 : 5-1 : 20 refer to ratios of ASC : DC. iDC: immature dendritic cell; Dex: dexamethasone control; mDC: mature dendritic cell; ASC: adipose tissue-derived stromal cell. Significant differences from mDC (adjusted *p* ≤ 0.05) marked with an asterisk (∗). *n* = 20, resulting from 5 ASC donors and 4 DC donors.

The presence of growth factors and trophic analytes was increased in response to escalating doses of ASC (Figure 5, Supplementary Table 1). Collectively, the concentrations of fibroblast growth factor 2 (FGF2), placental growth factor (PlGF), hepatocyte growth factor (HGF), and leukemia inhibitory factor (LIF) were significantly higher in ASC coculture than in mDC, while platelet-derived growth factor (PDGF-BB) was reduced. The relative presence of FGF2 was very low in iDC (0.32 ± 0.01), below that in mDC for the dexamethasone control (0.92 ± 0.01), but elevated in response to ASC at ratios of 1 : 20 (1.29 ± 0.04), 1 : 10 (1.60 ± 0.07), and 1 : 5 (2.06 ± 0.11). No significant difference in HGF concentration between mDC and the dexamethasone control (0.95 ± 0.02) was observed, but for ASC ratios of 1 : 20 (1.68 ± 0.06), 1 : 10 (2.02 ± 0.08), and 1 : 5 (2.39 ± 0.11), concentrations were increased. iDC remained low (0.39 ± 0.02). The increase in LIF was slight but consistent, with significant differences in 1 : 20 (1.07 ± 0.01), 1 : 10 (1.12 ± 0.01), and 1 : 5 (1.19 ± 0.02). The dexamethasone control (0.93 ± 0.01) was lower than mDC levels, with iDC still lower (0.68 ± 0.02). PlGF from iDC (0.75 ± 0.02) and the dexamethasone control (0.94 ± 0.01) went through no statistical difference at 1 : 20 (0.99 ± 0.02) to increased concentrations at 1 : 10 (1.19 ± 0.04) and 1 : 5 (1.56 ± 0.08). In contrast to the other analytes, the relative concentration of PDGF-BB plummeted in 1 : 20 (0.23 ± 0.01), 1 : 10 (0.20 ± 0.01), and 1 : 5 (0.18 ± 0.01), whereas the dexamethasone control (1.11 ± 0.02) was elevated and iDC (0.93 ± 0.03) was insignificantly different from mDC.

### 3.4. Cytokines Correlate with Trophic Factors

**Figure 6:**
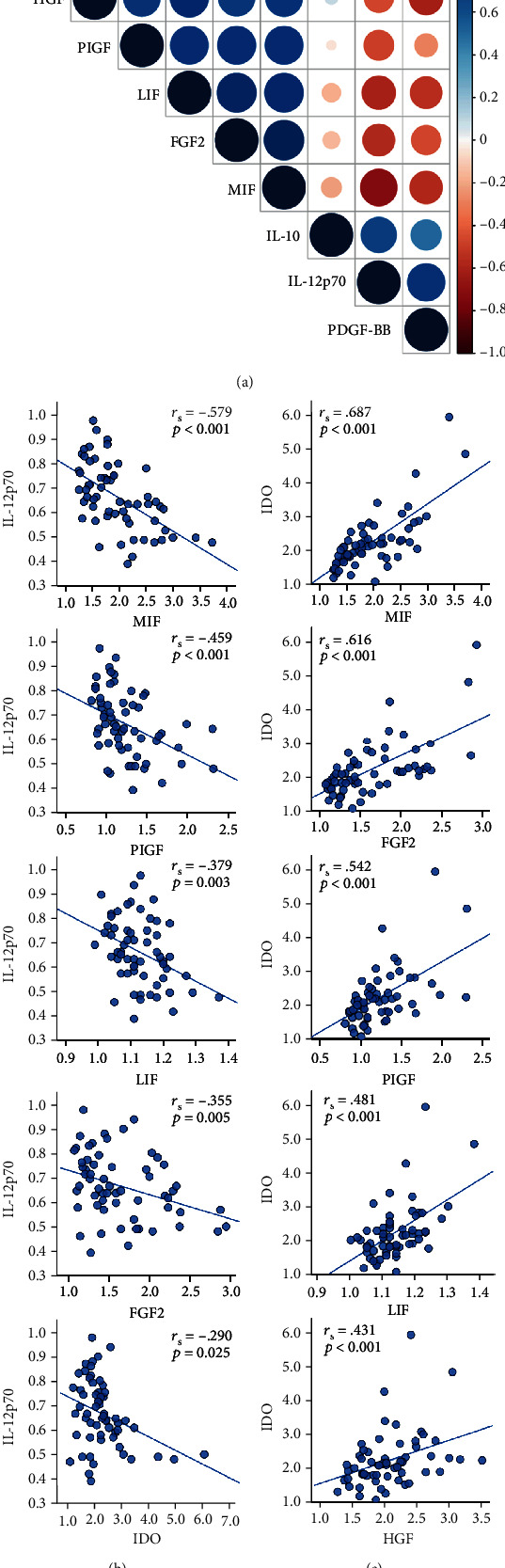
Correlations of relative concentration of analytes in cocultures of dendritic cells and ASC. (a) Correlation matrix of investigated factors. (b) IL-12p70 correlates inversely with ASC-associated factors, while the concentration of IDO correlates with the presence of trophic factors (c). Spearman's rho; *n* = 60, resulting from 5 ASC donors, 4 DC donors, and 3 cell ratios.

A central effector cytokine produced by mDC is IL-12p70. By correlating IL-12p70 with the presence of other analytes, it became evident that an inverse relationship to ASC-associated factors could be established (Figure 6(a)). This was particularly the case for the negative correlation with MIF (*r*_s_ = −0.579, *p* < 0.001) and PlGF (*r*_s_ = −0.459, *p* < 0.001), to a lesser extent for LIF (*r*_s_ = −0.379, *p* = 0.003) and FGF2 (*r*_s_ = −0.355, *p* = 0.005), and weakly for IDO (*r*_s_ = −0.290, *p* = 0.025).

Contrary to IL-12p70, IDO has gained much attention as a putative mechanism of action underlying MSC immunomodulatory function as well as a product of tolerogenic DC. The concentration of IDO correlated well with the factors attributed to the presence of ASC (Figure 6(b)). The best correlations were observed for MIF (*r*_s_ = 0.687, *p* < 0.001), FGF2 (*r*_s_ = 0.616, *p* < 0.001), PlGF (*r*_s_ = 0.542, *p* < 0.001), LIF (*r*_s_ = 0.481, *p* < 0.001), and HGF (*r*_s_ = 0.431, *p* < 0.001).

## 4. Discussion

In this study, the effect of ASC on maturation of monocyte-derived DC was investigated. We observed for the first time that cocultivation with ASC caused a change in the phenotypic profile of mDCs towards a less mature state, while simultaneously creating a trophic microenvironment.

The level of CD11c, a common marker for DC, was reduced in mDC cultures, which received maturation stimuli (LPS and IFN-*γ*), consistent with activation [10], yet more profoundly on exposure to ASC. In all instances, the generated DCs were CD11-positive, and gating on this population eliminated the risk of including ASC in the subsequent analysis. The expression of HLA-DR as well as costimulatory molecules CD40, CD80, and CD86 was reduced by escalating doses of ASC, exceeding the effect of the immunosuppressive dexamethasone control, which has been extensively utilized to generate tolerogenic DCs [11, 12]. These findings add to the evidence of ASC being able to downregulate maturation markers typically associated with activation of DCs. While the markers are in line with a common DC phenotype, it does not discriminate between subtypes or activation pathways affected by ASC, which is beyond the scope of the present study but constitutes an interesting subject for further research.

Interestingly, the expression of CD83 was increased in response to ASC but not dexamethasone. In a previous study of ASC and iDC coculture, we found decreased levels of CD80, CD86, and HLA-DR but did not investigate CD83 [13]. In addition to this, Li et al. reported decreased levels of CD80 and CD86 when MSC isolated from bone marrow were included during iDC maturation with LPS, yet no inhibition of CD83 expression, whereas MSC present during differentiation from monocytes to iDC did present decreased levels of CD83 [14]. Furthermore, CD83 was not increased after LPS-induced maturation when DC had been cocultured with ASC or MSC during differentiation from monocytes [15]. The expression of CD83 is upregulated during maturation of DCs; however, CD83 may play a role in tolerance as Kryczanowsky et al. identified a CD83^bright^ subpopulation as having superior immunosuppressive capabilities. Despite the heightened suppression, it should be noted that the CD83^bright^ subpopulation expressed CD80, CD86, and HLA-DR at levels comparable to mDC and that no relevant IL-10 secretion could be observed [16]. In the present study, the increased expression of CD83 was not accompanied by elevated levels of HLA-DR, CD40, and CD80 nor CD86, which does not indicate maturation. The expression of PD-L1 was not abrogated by ASC, and as PD-L1 expression on DC is important in suppressing T cell responses, correlating with the induction of tolerance to transplants [17], this suggests a preserved immunomodulatory function.

The measured levels of cytokines and soluble factors present in the coculture supernatant represent a sum of secretion, and the source can therefore not be attributed to DC or ASC, yet the results can shed light on the induced local milieu. An alternative approach would be to block the secretion of factors and detect the intracellular presence by flow cytometry; however, this does not necessarily reflect the actual composition of the factors released to the extracellular environment. By including intracellular markers, the opportunity to sort cell populations for functional assays is lost. Likewise, additional activation by, e.g., phorbol 12-myristate 13-acetate (PMA) and ionomycin could be included to potentiate the production and secretion of factors but may in turn divert further from the true polarization of the medium and could even affect ASC function leading to misinterpretation of modes and mechanisms of action underlying ASC-DC interactions. We therefore sought to detect and quantify the presence of soluble factors.

Activation of DC causes upregulation of IL-12, which induces polarization of T helper cell (Th) towards a Th1 phenotype important for cell-mediated immune responses [18]. In coculture with ASC, the presence of IL-12 was reduced in a dose-dependent manner and almost halved at the ratio of 1 : 5, providing evidence for immunosuppression in line with other studies [19–21], suggestive of less polarization towards a Th1 phenotype. The reduction in IL-12 was not accompanied by an increase in IL-10, which remained constant. The level of IL-10 in DC and MSC coculture has been reported as both increased and no effect of MSC coculture [13, 15, 22]. The discrepancy could be due to differences in differentiation protocols, maturation stimuli, timing of coculture, and differences in sources of MSC. Collectively, the reduction in IL-12 in combination with preserved IL-10 is suggestive of a less mature phenotype and could infer a hypoimmunogenic response following ASC treatment.

An often-highlighted immunosuppressive factor derived from ASC is IDO [20, 23, 24]. As the rate-limiting catabolic enzyme of the essential amino acid tryptophan, IDO restricts cell proliferation by limiting access to a fundamental nutrient, yet the effect is not limited to tryptophan depletion as the degradation products, kynurenines, have central immunoregulatory functions [25]. While IDO may originate from DC, licensing of MSC with IFN-*γ* highly increases IDO expression [26], and as ASCs are exposed to IFN-*γ* during the maturation of iDC to mDC, it is likely the cause for the increased expression in response to the ASC dose. Regardless of the source, the elevated level of IDO is central, as it has been reported to convert mDC to tolerogenic antigen-presenting cells, thereby suppressing T cell proliferation and inducing immune suppression, while promoting activation of regulatory T cells [27]. By promoting tolerance, the immune responses are impaired, which is crucial in limiting reactivity to self-antigens. Aside from its effects on DC, IDO has extensive effects on several types of leukocytes, including the T cell [28], B cell [29], and NK cell [30], which illustrates a wide spanning ability to modulate immune responses.

By having proinflammatory and immunoregulatory properties, the elevated level of MIF in ASC cocultures is an interesting finding. MIF can inhibit cell cycle arrest and apoptosis, lead to proliferation, and increase transcription of inflammatory genes [31]. However, recent evidence suggests that MIF can skewer DCs towards a tolerogenic phenotype [32]. As the concentration of IL-12 is decreased in the conditions that give rise to elevated MIF (correlation *r*_s_ = −0.579, *p* < 0.001), it is likely not attributed to increased inflammatory activity and may pose a potential mechanism of interaction. In light of these findings, it is possible that the presence of MIF can induce a more tolerogenic DC phenotype, which may result in dampened T cell responses.

While the regenerative potential of ASC may be linked to immunosuppressive function, other areas of interest should not be neglected, e.g., regenerative functions. If inflammatory stimuli cause increased immunosuppressive activity of ASC, this effect could potentially occur at the expense of growth factor production. However, we found that the ability of ASC to provide trophic growth factors persisted, exemplified by the elevated levels of FGF2, HGF, PlGF, and LIF in response to increasing cell ratios. Besides having properties related to antifibrosis, angiogenesis, and regeneration [33–36], the factors have implications on immune function. For instance, MSC-derived PlGF inhibits the maturation of DC and induces M2 polarization in macrophages [37], and LIF impairs maturation of DC [38], inhibits lymphocyte proliferation [39], and induces regulatory T cells which in turn produce LIF [40]. Separately, these factors and interactions represent possible mechanisms of action, with pleiotropic effects depending on the target cell.

Considerable levels of PDGF-BB were detected in cultures of iDC, mDC, and dexamethasone-treated mDC, whereas the addition of ASC almost completely depleted the concentration, regardless of the number of ASCs. PDGF-BB is involved in wound healing and angiogenesis by constituting a potent growth factor capable of inducing proliferation of mesenchymal cells [41]. Given the fact that ASCs are expanded in growth medium supplemented with hPL, which is rich in PDGF-BB, and rely on it for proliferation [42], it comes as no surprise that ASC rapidly and efficiently depletes the medium for this growth factor. Whether this interesting finding can be exploited, by reducing the abundance of PDGF-BB or similar in a pathological setting, has not been investigated.

The abundance of pleiotropic factors associated with ASC was inversely correlated with the concentration of the proinflammatory cytokine IL-12, while the amount of IDO correlated well with the presence of these factors. Together, this illustrates how ASC can interact with DC to generate a more tolerogenic environment and that factors such as MIF, PlGF, and LIF contribute to the process. However, as the concentrations of several factors are closely linked to the ratio of ASC, it cannot be ruled out that these factors merely reflect the number of ASC and that the high correlation is dependent on cell counts rather than actual inhibitory function of the factors *per se*, with other underlying mechanisms at play.

Regardless of the DC or ASC donor, the suppression of maturation exhibited a strong response, which emphasizes the robustness of ASC immunomodulation, often surpassing the effect of dexamethasone, a known immunosuppressant and inducer of tolerogenic DC. By extending the analyses from immunological factors to factors associated with regeneration, the model offers a more complete characterization of ASC function in a specific setting. To completely elucidate how the changes in the DC induced by ASC coculture could lead to downstream effect on other leukocytes, the DC would have to be collected and subsequently cocultured with responder cells. This, however, was not the scope of the current study.

The rigorous testing of ASC on DC function following cocultivation shed light on multiple potential mechanisms of action, and the model serves as a strong tool for further exploration. Several factors have been identified as being negatively correlated with IL-12p70, which may eventually serve as surrogate markers. For future elucidation of ASC-induced DC tolerogenicity, inhibition of, e.g., IDO or MIF would be a relevant starting point.

In summary, we have described how ASCs affect the maturation of monocyte-derived DC by altering the phenotypical profile of classical markers and by reducing the production of the proinflammatory cytokines while increasing the concentration of immunosuppressive factors. In addition to pushing the local environment towards immunomodulation, ASC cocultivation provided a regenerative environment with pleiotropic growth factors. While the inclusion of dexamethasone caused a decrease in DC maturation markers, the extent did not match higher ratios of ASC and dexamethasone treatment did not induce the same regenerative environment based on the observed increase in growth factors. To our knowledge, this is the first time that these factors have been assayed in an ASC-DC coculture model. Collectively, these findings shed light on the multifaceted role of ASC in immunomodulation and add to a growing body of evidence, where the described assay plays an important role in further elucidation of the potential mechanisms of action.

The ASC cell product employed in the present study has been manufactured for clinical use. To closely resemble a therapeutic setting, five ASC donors were included and tested against four DC donors, yielding results independent of possible mismatches in compatibility. The addition of ASC efficiently reduced the maturation of DC, displaying a dose-response relationship. The immunomodulatory effects were observed simultaneously with elevated levels of trophic factors, underlining the ability of ASC to influence multiple processes at once, in this case affecting immune function and regeneration similarly. For pathologies with etiologies under dual influence of inflammation and reduced component of healing, such as chronic wounds or heart failure, the multifarious abilities of ASC could prove to be indispensable for the therapeutic outcome. Considering the frequent inclusion of new potential mechanisms of actions supporting ASC function, the described dendritic cell coculture model constitutes a precious tool for elucidating factors, testing cell products, and documenting potency in an *in vitro* assay.

## 5. Conclusions

ASC produced and cryopreserved for clinical use robustly inhibited the maturation of DC while simultaneously providing trophic factors. This provides evidence that ASC therapy can function regeneratively while evading or suppressing the immune response, which is important in allogeneic therapy.

## Data Availability

The data used to support the findings of this study are included in the article and within the supplementary information files.

## Supplementary Materials

Supplementary Materials Supplementary Table S1 describes the absolute concentrations of cytokines and growth factors.
