# Supplementary material for: Adipose Tissue-Derived Stromal Cells Induce a Highly Trophic Environment While Reducing Maturation of Monocyte-Derived Dendritic Cells
Source: Stem Cells Int. 2020 Oct 26;2020:8868909. doi: 10.1155/2020/8868909 (PMC7607274; doi:10.1155/2020/8868909)
Supplement: Supplementary Materials — Supplementary Table S1 describes the absolute concentrations of cytokines and growth factors. [file 8868909.f1.docx]

Supplementary Table 1. Concentrations of growth factors and cytokines

|  | iDC (pg/ml) | | Dex (pg/ml) | | 1:5 (pg/ml) | | 1:10 (pg/ml) | | 1:20 (pg/ml) | | mDC (pg/ml) | |
| --- | --- | --- | --- | --- | --- | --- | --- | --- | --- | --- | --- | --- |
| IL-12p70 | 676.15 | ± 16.60 | 5,040.87 | ± 800.51 | 7,699.99 | ± 869.17 | 9,653.77 | ± 1,127.50 | 11,344.38 | ± 1,378.23 | 14,513.39 | ± 2,070.83 |
| IL-10 | 15.13 | ± 0.31 | 47.03 | ± 4.36 | 70.87 | ± 3.78 | 70.42 | ± 3.65 | 70.96 | ± 3.40 | 80.45 | ± 5.73 |
| MIF | 23,670.96 | ± 1,484.87 | 21,776.46 | ± 1,693.97 | 50,623.97 | ± 1,520.64 | 35,790.60 | ± 1,519.51 | 28,509.60 | ± 1,261.13 | 19,740.88 | ± 1,029.55 |
| IDO | 1,887.33 | ± 67.37 | 9,047.50 | ± 1,015.52 | 17,797.83 | ± 1,521.44 | 14,886.08 | ± 1,032.41 | 12,550.03 | ± 1,077.97 | 6,980.75 | ± 844.61 |
| FGF2 | 24.31 | ± 0.69 | 69.58 | ± 1.63 | 155.71 | ± 8.41 | 121.11 | ± 6.54 | 97.51 | ± 4.06 | 75.49 | ± 1.30 |
| HGF | 156.66 | ± 10.54 | 404.90 | ± 41.51 | 980.86 | ± 60.46 | 841.26 | ± 59.04 | 702.70 | ± 50.23 | 432.29 | ± 46.94 |
| LIF | 191.64 | ± 6.06 | 263.04 | ± 4.02 | 336.74 | ± 5.04 | 318.07 | ± 3.65 | 303.24 | ± 2.59 | 283.44 | ± 2.02 |
| PlGF | 19.30 | ± 0.63 | 24.15 | ± 0.46 | 40.14 | ± 2.21 | 30.42 | ± 1.20 | 25.48 | ± 0.70 | 25.62 | ± 0.28 |
| PDGF-BB | 930.47 | ± 21.70 | 1,118.25 | ± 32.81 | 186.10 | ± 12.41 | 203.22 | ± 14.24 | 238.05 | ± 16.74 | 1,015.85 | ± 46.26 |

Absolute concentrations in pg/ml of analytes ± standard error, according to condition. 1:5-1:20 refer to ratios of ASC:DC. iDC: immature dendritic cell, Dex: Dexamethasone control, mDC: mature dendritic cell, ASC: Adipose tissue-derived Stromal Cell.
